# The Impact of Parents' Intelligence Mindset on Math Anxiety of Boys and Girls and the Role of Parents' Failure Beliefs and Evaluation of Child's Math Performance as Mediators

**DOI:** 10.3389/fpsyg.2022.687136

**Published:** 2022-06-27

**Authors:** Fang Xie, Xiangfei Duan, Xuelian Ni, Lina Li, Libin Zhang

**Affiliations:** ^1^School of Education, Chongqing Normal University, Chongqing, China; ^2^School of Psychology and Mental Health, North China University of Science and Technology, Tangshan, China; ^3^School of Education, China West Normal University, Nanchong, China; ^4^Collaborative Innovation Center of Assessment for Basic Education Quality, Beijing Normal University, Beijing, China

**Keywords:** parent's failure mindset, parent's intelligence mindset, gender difference, math anxiety, Chinese students

## Abstract

This study aimed to examine the relationship between parents' intelligence mindset and children's math anxiety and the mediating role of parents' failure mindset and evaluations of their child's math performance. A total of 419 Chinese students (196 boys and 223 girls) and their parents were recruited to complete a series of questionnaires on topics such as math anxiety, parent's failure mindset, parent's intelligence mindset, and parents' evaluations of their child's mathematical performance. The results revealed that parents' intelligence mindset was not correlated with children's math anxiety. However, parents' intelligence mindset indirectly predicted children's math anxiety through the chain-mediated role of parents' failure beliefs and parents' evaluations of their child's math performance. Further, sex differences were found through a multigroup analysis, which showed a chain-mediated effect between parents' intelligence mindset and girls' math anxiety.

## Introduction

Math anxiety is the feeling of dread elicited by dealing with math-related situations (Suárez-Pellicioni et al., 2016). This type of anxiety typically appears in the first grade (Gunderson et al., 2012; Ramirez et al., 2013) and gradually increases by grade (Krinzinger et al., 2009), often to prevalence levels that are beyond our expectations (Suárez-Pellicioni et al., 2016; Ahmed, 2018). For instance, 93% of Americans have reported a negative math experience (Furner and Duffy, 2002). Data from the 2012 Program for International Student Assessment (PISA) revealed that more than 61% of 15-year-old students across 65 countries expressed varying levels of math anxiety, with 30% reporting high math anxiety (Suárez-Pellicioni et al., 2016; Ahmed, 2018).

Math anxiety is a major obstacle to success in mathematics. Individuals with high math anxiety have low math self-efficacy, low math motivation, negative math learning attitudes, and low math achievement (Ashcraft and Krause, 2007; Casad et al., 2015). Those with math anxiety often avoid math-related, and STEM careers (Ahmed, 2018), as mathematics is considered a gateway field for other STEM disciplines (Casad et al., 2015). Results of an fMRI study revealed that pain-related brain regions (bilateral dorso-posterior insula) are activated for adults with high math anxiety when anticipating an upcoming math task (Lyons and Beilock, 2012). Hence, individuals with high math anxiety regard math as a dreaded event, which activates the pain network. These findings suggest that math anxiety triggers not only psychological suffering but also generates physical pain.

### The Relationship Between Parents' Intelligence Mindset and Child's Learning in Math

Based on an individual's beliefs about intelligence, Dweck (2009) identified two mindsets, fixed and growth. In general, people with a fixed mindset believe that intelligence is innate. They attribute success to being smart and tend to avoid challenges or often fail to meet their potential (Dweck, 2009, 2017; Yeager and Dweck, 2020). Conversely, people with a growth mindset believe that intelligence is changeable (Dweck, 2009, 2017; Yeager and Dweck, 2020). Previous studies have demonstrated that students' intelligence mindsets are correlated with their math learning. Jiang et al. (2019) examined 1,064 high school students and found that their fixed mindset was correlated with their mathematical academic self-efficacy and negative academic emotions (shame and boredom) about math. Su et al. (2021) suggested that a growth mindset was positively related to primary school students' math self-efficacy and math grades. Another study demonstrated that a growth mindset contributed to junior high school students' math performance (Blackwell et al., 2007). Thus, students' intelligence mindsets affect math performance and math attitude for students in different grade levels.

Parents' intelligence mindset has an important effect on children's intelligence mindset (Gunderson et al., 2012, 2013), which has been found to relate to students' math learning. Some studies further explored the relationship between parental intelligence mindset and children's math learning, but the results were not consistent. For example, Cheng et al. (2017) found that parents' growth mindset affected children's math self-efficacy. Further, parents' growth mindset had a larger effect on girls' math self-efficacy than boys'. Math self-efficacy has been identified as an important factor for math anxiety (Akin and Kurbanoglu, 2011; McMullan et al., 2012). Therefore, we assumed that parents' growth mindset would have a larger effect on girls' math anxiety than that of boys.

### Parents' Failure Beliefs and Parents' Evaluation of Child's Math Performance as Mediators

#### Parents' Failure Beliefs as Mediators

Failure beliefs refer to individuals' views about failure, whether it is enhancing or debilitating (Haimovitz and Dweck, 2016). As shown above, an individual's intelligence mindset determines their view of failure. Individuals with a fixed mindset pursue success and avoid failure. Because success is considered a performance of ability, and failure is considered a performance of incompetence. As a result, people with a fixed mindset are more likely to view failure as debilitating. In contrast, individuals with a growth mindset believe that ability can be changed through efforts, and failure is an opportunity to help them develop their abilities. Therefore, people with a growth mindset are more likely to view failure as enhancing their abilities (Dweck, 2009, 2017; Yeager and Dweck, 2020). It can be seen that an individual's failure beliefs reflect the individual's intelligence mindset in a failure situation. A previous study has shown that students' intelligence mindset predicted their failure beliefs (Su et al., 2021).

However, compared to parents' intelligence mindset, it has been suggested that a parent's failure mindset is obvious to the child, as it is accompanied by specific externalized behaviors and directly impacts the development of the child's intelligence mindset (Haimovitz and Dweck, 2016). For example, Haimovitz and Dweck (2016) found that a parent's failure-as-debilitating mindset led to elementary students' fixed mindset. Tao et al. (2022) also found that students' perceptions of their parents' failure mindset predicted college students' intelligence mindset 1 year later. As shown above, children's growth mindsets may contribute to their math self-efficacy. Considering the close relationship between math self-efficacy and math anxiety (Akin and Kurbanoglu, 2011; McMullan et al., 2012), parents' failure beliefs may be associated with students' math anxiety. Thus, parents' failure beliefs may play a mediating role between parents' intelligence mindset and their child's math anxiety.

#### Parents' Failure Beliefs and Parents' Evaluation of Child's Math Performance as Chain-Mediator

In addition, parents' evaluations of their child's mathematical performance may play a mediating role between parents' failure mindset and the math anxiety of the girl child. A previous study reported that a parent's failure mindset leads to different reactions to a child's failure (Haimovitz and Dweck, 2016). Parents who believe that failure is debilitating have ability-oriented behaviors (Haimovitz and Dweck, 2016; Dweck, 2017). Moreover, in some countries like China, the stereotype that boys have a stronger math ability than girls still exists (Dong, 2019). Specifically, parents with a failure-is-debilitating mindset tend to give girls a lower evaluation of mathematical ability when compared to boys. Previous studies also have reported that girls are more concerned with a parent's evaluation than boys (Au and Harackiewicz, 1986; Henderlong Corpus and Lepper, 2007). This may be because the social orientation of females is dependent and interpersonally aware, while that of males is independent and achievement-focused (Deci and Ryan, 1985). Parents' lower evaluations of girls' math abilities may deepen a girl's belief that girls have no talent in mathematics, which then increases girls' math anxiety. Therefore, we assumed that a parent's failure mindset might affect a girl's math anxiety through the parent's evaluation of their child's mathematical performance.

### Influence of Gender

Gender difference is always a hot issue in math anxiety. Many studies have reported that females have higher math anxiety than males (Else-Quest et al., 2010; Ramirez et al., 2013; Xie et al., 2019). Previous studies have explored individual and environmental reasons for these gender differences. Individual factors associated with females' higher math anxiety, when compared to males, include lower spatial ability (Maloney et al., 2012; Wang, 2020), low self-esteem (Xie et al., 2019), low mathematical self-concept (Bieg et al., 2015), and low perceived controllability about math (Zirk-Sadowski et al., 2014). Environmental factors include gender stereotypes (Bieg et al., 2015), the teacher's math anxiety (Beilock et al., 2012; Gunderson et al., 2012), and parents' behaviors.

Although no studies have demonstrated that parents' intelligence mindset and parents' failure beliefs are different for son and daughter, research indicates that parents often believe that boys have more talent in math than girls (Gunderson et al., 2012; Bieg et al., 2015; Li and Bates, 2018), and place a stronger emphasis on the math ability of their sons than that of their daughters (Stoet et al., 2016; Cui et al., 2019). In addition, research also shows that parents attribute a boy's success to talent and failure to lack of hard work, while they attribute a girl's success to hard work and failure to lack of talent (Gunderson et al., 2012). This kind of parental feedback could impact the development of girls' fixed mindsets in math (Gunderson et al., 2013), which could explain findings like those of Cheng et al. (2017), who found that boys were more likely to report a growth mindset than girls. All these studies show that parents' ability beliefs, attribution, and evaluation of math performance are different for boys and girls. Thus, the relationships between parents' intelligence mindset, parents' failure beliefs, parents' evaluation, and child's math anxiety may differ for boys and girls.

### The Present Study

Currently, there is no research that explores the relationship between the intelligence mindset and math anxiety, which could be a critical factor for students' math learning ability. Considering parental beliefs affect students' intelligence mindsets, the present study aimed to explore the relationship between math anxiety, parents' intelligence mindset, and parents' failure mindset, and whether these relationships change with gender.

**Figure 1:**
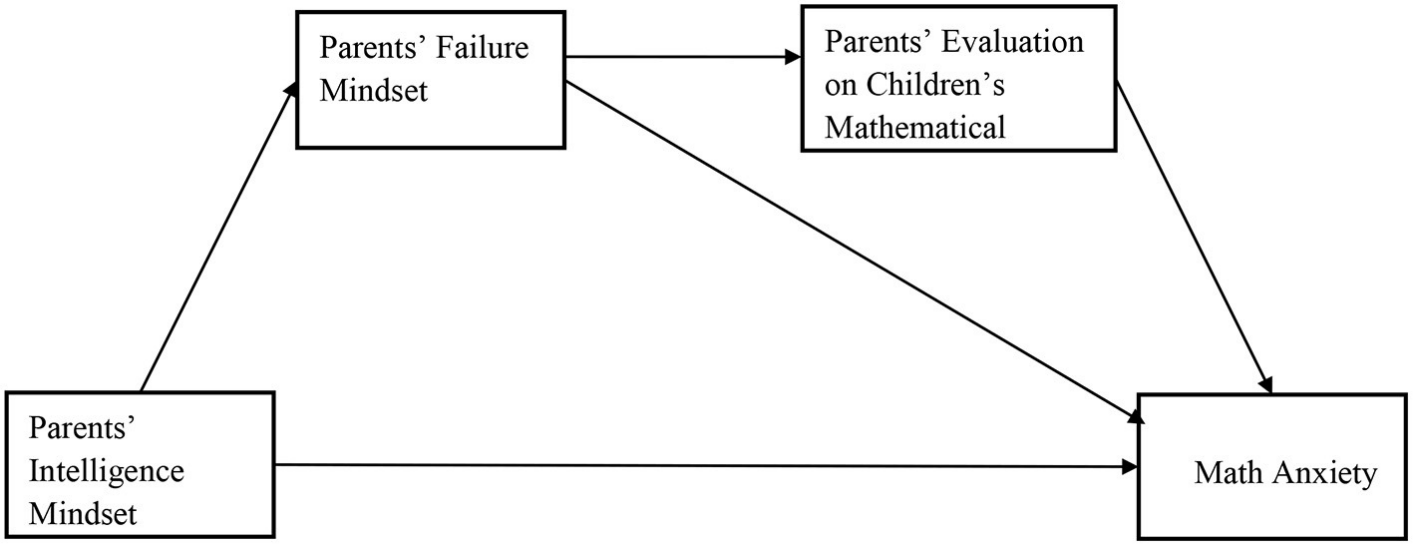
The hypothesis model of the relationship between variables.

The study had three main objectives: first, to examine the relationship between parents' intelligence mindset and students' math anxiety; second, to explore the mediating role of parents' failure beliefs in the relationship between parents' intelligence mindset and students' math anxiety; and third, to explore whether the relationship between parents' intelligence mindset and children's math anxiety may be mediated by the chain-mediating role of parents' failure beliefs and their evaluation about their child's math performance. Based on our hypotheses, the current model was proposed (see Figure 1).

## Methods

### Participants

A total of 419 students from Grades 4 to 9 (ages ranging from 8 to 17 years old) were selected from three public schools in <city>Tangshan</city>, China. There were 83 students in Grade 4 (*M age* = 9.80, *SD* = 0.84), 74 students in Grade 5 (*M age* = 10.74, *SD* = 0.64), 78 students in Grade 6 (*M age* = 11.71, *SD* = 0.54), 66 students in Grade 7 (*M age* = 12.79, *SD* = 0.59), 35 students in Grade 8 (*M age* = 13.69, *SD* = 0.83), and 74 students in grade 9 (*M age* = 14.96, *SD* = 0.80). Furthermore, there were 196 boys (*M age* = 11.91, *SD* = 1.92) and 223 girls (*M age* = 12.23, *SD* = 1.92). Student questionnaires were immediately filled out and collected, while parent questionnaires were brought back by the students and collected the next day. Of the questionnaires, 64.4% were completed by the mother, while 33.7% were completed by the father, and eight participants did not include their parents. Most parents (65.6%) only had a high school degree or lower. This study was approved by the Ethics Committee of North China University of Science and Technology. All participation in this study was voluntary, and informed consent was obtained from parents in advance.

### Measures

#### Parent's Intelligence Mindset

The Growth Mindset scale for adults (Blackwell et al., 2007) (Cronbach's α = 0.90; two-week test-retest reliability: *r* = 0.77) was translated into Chinese. The translated version was tested among 474 Chinese parents, and the reliability was 0.76. Therefore, four original items were retained: two items representing the fixed intelligence mindset (“*You have a certain amount of intelligence, and you really can't do much to change it.” “You can learn new things, but you can't really change how intelligent you are.”*); the two other items representing a growth intelligence mindset (“*No matter how much intelligence you have, you can always change it quite a bit.” “You can always greatly change how intelligent you are.”*). The items used a Likert-type scale from 1 (*disagree strongly*) to 6 (*agree strongly*). The scores for the growth intelligence mindset were reverse coded. Higher summed scores indicated a more fixed view of intelligence. Cronbach's α for the present study was 0.64.

#### Parent's Failure Mindset

The Failure Mindset Scale was translated into Chinese (Haimovitz and Dweck, 2016; α = 0.88). This scale contained six items. The first three items represent failure as an incentive (e.g., “*experiencing failure facilitates learning and growth*”), while items 4, 5, and 6 represent failure as a setback (e.g., “*experiencing failure inhibits my learning and growth*”). The items were rated on a Likert-type rating scale from 1 (*disagree strongly*) to 6 (*agree strongly*). The scores for items 1, 2, and 3 were reverse coded. Higher summed scores indicated a failure-is-debilitating mindset. The translated version among study participants exhibited reasonable reliability (α = 0.76).

#### Math Anxiety

The Abbreviated Math Anxiety Scale (AMAS; Hopko et al., 2003) was used to measure levels of math anxiety. This also was translated into Chinese. The AMAS is the shortest known valid math anxiety scale with only nine items, but it has been shown to be as effective as the longer Math Anxiety Rating Scale (MARS; Hopko et al., 2003) (Cronbach's α = 0.90; two-week test-retest reliability: r = 0.85; convergent validity of AMAS and MARSR, r = 0.85). The items on this scale (e.g., “*thinking about an upcoming math test one day before*”) had response options from 1 (not anxious at all) to 5 (very anxious). Higher summed scores indicated greater math anxiety (α = 0.86).

#### Parent's Evaluation of Child's Mathematical Ability

Parents were instructed to “Please evaluate your child's mathematical ability.” The response options ranged from 1 (*very bad*) to 6 (*very good*).

## Results

### Descriptive Analysis and Intercorrelations

The t-test was conducted to test the effect of gender on math anxiety, parents' failure mindset, parents' intelligence mindset, and parents' evaluations of their child's mathematical performance (see Table 1). Results revealed a statistically significant gender difference for math anxiety (t [1, 417] = −3.280, *p* = 0.001, Cohen's d = −0.323), in which girls *(M* = 24.16, *SD* = 5.74) reported higher math anxiety than boys (*M* = 22.13, *SD* = 6.77). In addition, parents' evaluation of child's math performance showed significant gender difference (t [1, 417] = 2.202, *p* = 0.028, cohen's d = 0.218). Parents reported higher evaluation of boys' math performance (*M* = 3.88, *SD* = 1.15) than of girls' math performance (*M* = 3.62, *SD* = 1.23).

**Table 1:** Descriptive statistics of variables on gender.

**Variables**	**Boys (*n* = 196)**	**Girls (*n* = 223)**	**t**	**Cohen's d**
1. Students' math anxiety	22.13 ± 6.77	24.16 ± 5.74	−3.280^**^	−0.323
2. Parents' failure mindset	11.94 ± 4.29	11.87 ± 4.31	0.170	0.016
3. Parents' intelligence mindset	11.13 ± 3.65	11.29 ± 3.66	−0.454	−0.044
4. Parents' evaluation of children's math performance	3.88 ± 1.15	3.62 ± 1.23	2.201^*^	0.218

* *p < 0.01*,

** *p < 0.001*.

Table 2 presents correlations among the variables. Results showed that parents' intelligence mindset was significantly positively correlated with their failure mindset (*r* = 0.426, *p* < 0.001), and negatively correlated with parental evaluation of their child's math performance (*r* = −0.163, *p* = 0.001). Parents' failure mindset had a statistically significant negative correlation with parents' evaluations of their child's math performance (*r* = −0.139, *p* = 0.004), and a positive correlation with math anxiety (*r* = 0.128, *p* = 0.009). Parents' evaluation of their child's math performance had a statistically significant negative correlation with math anxiety (*r* = −0.305, *p* < 0.001).

**Table 2:** Descriptive statistics and intercorrelations.

**Measures**	**M ±SD**	**1**	**2**	**3**	**4**
1. Parents' intelligence mindset	11.22 ± 3.65	1			
2. Parents' failure mindset	11.91 ± 4.29	0.426^***^	1		
3. Parents' evaluation of children's math performance	3.74 ± 1.19	−0.163^**^	−0.139^**^	1	
4. Math anxiety	23.21 ± 6.32	0.065	0.128^**^	−0.305^***^	1

** *p < 0.01*,

*** *p < 0.001*.

### Structural Equation Modeling

**Figure 2:**
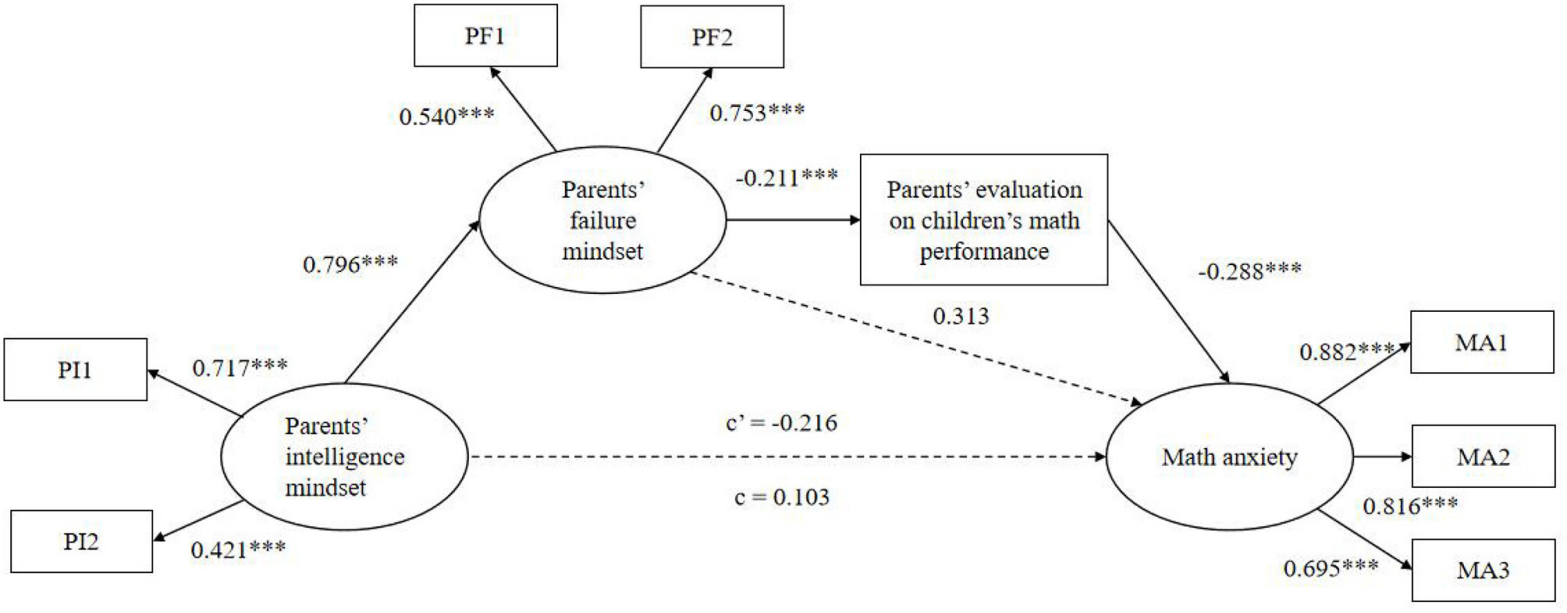
Full model; ***p < 0.001.

It was hypothesized that parents' intelligence mindset would be associated with students' math anxiety through parents' failure mindset and parents' evaluations of their child's mathematical performance. Structural equation modeling was used to test this hypothesis. Intelligence mindsets and failure mindsets were indexed using two separate items; math anxiety was indexed by three items using a factorial algorithm. A factor analysis was conducted, and the item load was arranged from high to low (Yang et al., 2010). There were nine items in the math anxiety questionnaire, with every three items representing a group. All the factor loadings ranged from 0.424 to 0.882 and were statistically significant, indicating that all measurement indicators could be well-explained by the latent variables. The tested model was found to be acceptable (Figure 2): χ^2^ (16) = 2.297, *p* = 0.002, *TLI* = 0.955, *CFI* = 0.974, *RMSEA* = 0.056, and *SRMR* = 0.033. The results revealed that parents' intelligence mindset was associated with parents' failure mindset (*b* = 0.796, *p* < 0.001); parents' failure mindset was associated with parents' evaluations of their child's math performance (*b* = −0.211, *p* < 0.001); and parents' evaluations of their child's math performance had a statistically significant association with students' math anxiety (*b* = −5.501, and *p* < 0.001).

To further test whether the mediating effect was significant, a bootstrapping analysis with 5,000 bootstrap samples was conducted. As shown in Table 3, the direct impact of parents' intelligence mindset on math anxiety was mediated by a chained-mediated role of parents' failure mindset and parents' evaluation of child's math performance (*b* = 0.102, *95% CI* [0.043, 0.214]). The direct effect of parents' intelligence mindset on their child's math anxiety was not significant (*b* = −0.454, *95% CI* [−3.611, 0.328]).

**Table 3:** Standardized total, direct, and indirect effects and 95% confidence intervals.

**Path**	**Estimate**	**Lower**	**Upper**
Direct effect: parents' intelligence mindset → child's math anxiety	−0.454	−3.611	0.328
Total indirect effect	0.624	0.091	4.625
Mediating effect			
M1: parents' intelligence mindset → parents' failure mindset → child's math anxiety	0.522	−0.015	4.457
M2: parents' intelligence mindset → parents' failure mindset → parents' evaluation on child's math performance child's math anxiety child's math anxiety	0.102	0.043	0.214
Total effect	0.170	−0.146	0.546

### Multi-Group Analysis of the Full Model

A multi-group analysis was conducted to explore whether the full model was equally valid across genders. As shown in Table 4, Model 1 has no constraint on the model parameters; Model 2 constrains the factor loadings to be equal; and Model 3 constrains the intercepts to be equal. All three models fit well. The results of the model comparison showed that the χ^2^ difference was nonsignificant between Models 1 and 2 as well as Model 2 and Model 3. And the difference in the fitting index (ΔTLI, ΔCFI, ΔRMSEA) between each of the models was <0.01, which showed that each equivalent model was established (Cheung and Rensvold, 2002). These results indicated that the structural relationships shown in Figure 2 did not have statistically significant differences between boys and girls.

**Table 4:** Multi-group analysis: boys vs. girls.

**Model**	**Specifications**	**χ^2^**	**df**	**TLI**	**CFI**	**RMSEA**	**Model comparison**	**χ^2^ diff**.	**df diff**.	***p***
1	Unconstrained	48.18	32	0.964	0.980	0.035				
2	Structural weights equal	51.99	36	0.969	0.980	0.033	1 vs. 2	3.81	4	0.432
3	Measurement residuals equal	66.41	44	0.964	0.972	0.035	2 vs. 3	14.42	8	0.071

**Figure 3:**
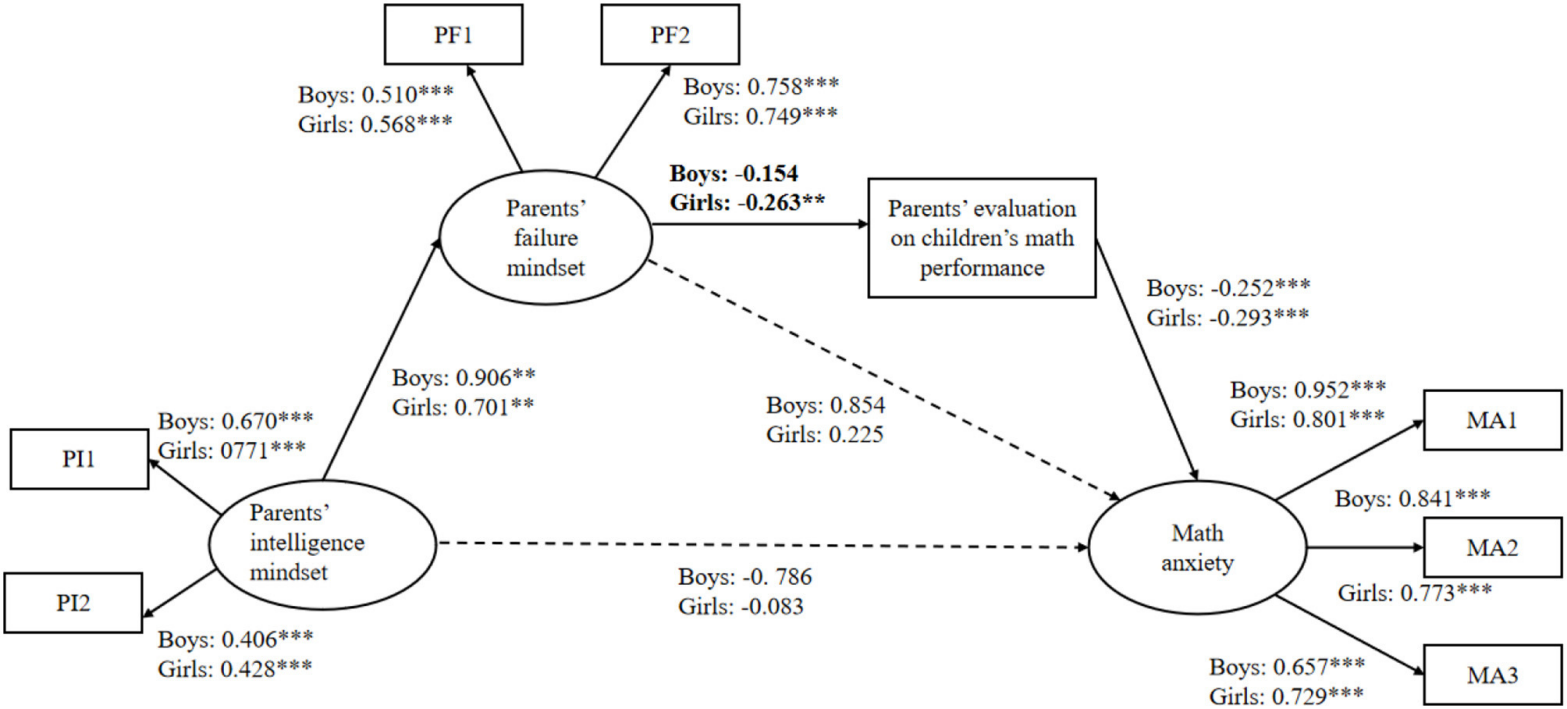
The SEM model for boys vs. girls; ***p < 0.001; **p < 0.01.

Further, comparing the unconstrained model (all of the structural pathways are estimated freely) and constrained model (all of the structural pathways are constrained to be equal) to test the difference in the mediation model between boys and girls, it was found that the unconstrained model fitted well, but the fitting index of the constrained model became worse. There was a significant difference between the unconstrained and constrained models (*p* = 0.037), and the difference in the fit index of the two models was >0.01 (ΔNFI = 0.021, ΔIFI = 0.022). Further comparison of the pathways showed that the relationship between parents' failure mindset and parents' evaluation of their child's math performance differed for boys and girls (see Figure 3). For boys, the direct path coefficient for parents' failure mindset on parents' evaluations of their child's math performance was not statistically significant (*b* = −0.154, *p* = 0.078). This result showed that the chain-mediated role of parents' failure mindset and parents' evaluation of their child's math performance was not found between parents' intelligence mindset and boys' math anxiety.

## Discussion

This study aimed to explore relationships among parents' intelligence mindset, parents' failure mindset, parents' evaluation of their child's math performance, and students' math anxiety. According to SEM results, parents' intelligence mindset did not predict students' math anxiety directly but did predict math anxiety through the chain-mediated role of parents' failure mindset and parents' evaluation of their child's math performance. Moreover, this chain-mediated effect was only established between parents' intelligence mindset and girls' math anxiety.

This study is the first to explore the relationship between parents' intelligence mindset and children's math anxiety. Results showed that parents' fixed mindset was not significantly correlated with children's math anxiety; however, parents' failure-is-debilitating mindset was positively related to children's math anxiety. This may be because parents' intelligence mindset is not always visible to children, but parents' failure beliefs are more visible as they are displayed in their reaction to their child's failure (Haimovitz and Dweck, 2016). Parents who hold the view that failure is debilitating focus more on a child's ability or performance (Haimovitz and Dweck, 2016). Therefore, when a child fails in mathematics, parents with a failure-is-debilitating mindset may blame the child for a lack of math ability, which leads to lower math self-efficacy and higher math anxiety in the child.

Although a direct correlation was not found between parents' intelligence mindset and children's math anxiety, parents' intelligence mindset predicted math anxiety through parents' failure mindset and parents' evaluations of their child's math performance. The intelligence mindset predicting the failure mindset has been demonstrated in some studies (Haimovitz and Dweck, 2016; Su et al., 2021). Based on Dweck's theory of intelligence mindset, individuals with different intelligence mindsets have varying attributions and attitudes toward failure (Dweck, 2009, 2017). People with a fixed mindset tend to believe that failure is debilitating as it threatens their ability. In turn, people with a growth mindset tend to believe that failure is enhancing as it contributes to their ability. Parents with a failure-is-debilitating mindset are more likely to focus on their child's performance or ability, while parents with a failure-is-enhancing mindset are more likely to focus on their child's learning. Therefore, parents with a failure-is-debilitating mindset put higher expectations on their child's math performance and give a lower evaluation of their child's actual math performance. Parents' low evaluation of math performance, when conveyed to the child, increases the child's math anxiety.

However, an important finding was that the chain-mediated role of parents' failure mindset and parents' evaluations of their child's math performance was only significant for girls. This result was consistent with our hypothesis. One explanation for this is gender stereotyping. Math has historically been thought of as a male domain, which leads some parents to believe that boys are better at math than girls. Parents' failure beliefs may deepen parents' lower evaluation of girls' math performance. To be specific, parents with a failure-is-debilitating mindset endorse performance-oriented behaviors, and they pay more attention to their child's math performance (Haimovitz and Dweck, 2016). Gender stereotypes in math convey a belief that boys are inherently better in math than girls. Thus, parents with a failure-is-debilitating mindset are more likely to give a lower evaluation of girls' math performance. In turn, parents' gender stereotyping in math makes them have a more positive view of boys' math performance. In other words, male-biased stereotypes in math may offset the influence of parents' failure beliefs on parents' evaluation of boys' math performance. As a result, parents' failure beliefs showed a significant correlation only with parents' evaluation of girls' math performance.

In addition, it was interesting to find that parental evaluation of their child's mathematical ability was negatively correlated with the child's math anxiety and that there was no gender difference. Previous studies have reported that girls are more concerned with their parents' evaluations than boys (Au and Harackiewicz, 1986; Henderlong Corpus and Lepper, 2007), but this may not be the case for mathematics. One explanation for this finding is that mathematics has been considered a male domain, which caused parental differences in evaluations for boys and girls. When boys have good mathematical performance, parents encourage them to pursue math-related careers. However, when girls have good mathematical performance, parents only hope that girls can maintain their performance and not fail in their college entrance examinations. When boys have poor mathematical performance, parents encourage boys to work harder. However, this kind of high mathematical expectation could increase math anxiety in boys. When girls have poor mathematical performance, parents may attribute this to a girl's mathematical ability being inferior to boys', reducing a girl's mathematical self-efficacy and thereby increasing a girl's math anxiety. Therefore, parents' evaluations of their child's mathematical performance could lead to higher math anxiety.

## Implications and Limitations

Environmental factors have a critical influence on the development of math attitudes (Gunderson et al., 2012). This study shows that parents' failure mindset has a negative indirect effect on girls' math anxiety. These results suggest that interventions would be beneficial for teaching parents to realize the benefits of failure and react more appropriately to their child's failure in mathematics. More process-directed praise should be encouraged (e.g., you must work hard to succeed) for both boys and girls, so they form an incremental mindset and show persistence after failure (Gunderson et al., 2012). Furthermore, an increasing number of studies show that there are no gender differences in mathematical performance (Else-Quest et al., 2010; Xie et al., 2019), which means that girls perform as well as boys in mathematics. Parents need to change their view that boys are more mathematically talented than girls and avoid making gender-biased statements in front of their children. However, most parents have no access to this type of information. Hence, school-based interventions are crucial. Schools should provide this information to parents via lectures or published scientific articles. Additionally, schools should provide mental health courses or activities to improve students' attitudes toward failure and help students develop a growth mindset.

Some limitations need to be noted. First, parents' intelligence and failure mindsets in the current study were general rather than specific to math. Mathematics is a relatively specialized field with strong gender stereotypes. However, the parents' mindsets we measured may not accurately represent parents' intelligence and failure mindsets for math. This limits our interpretation of the current results. Future research should consider domain-specific measures of parents' intelligence and failure mindsets for math. Second, the current research was based on a cross-sectional design, which generates biased estimates of mediation parameters. Because cross-sectional models do not allow statistical control for prior levels of M or Y in a mediation model, it fails to explore the influence of one measure on another over time (Maxwell and Cole, 2007). Thus, it needs to be stressed that the structural equation models used in our study could not reveal any causal relationships. Experimental, longitudinal, and intervention studies are needed in future to address causality. Third, the present study did have access to the student's actual mathematical performance. This prevented the investigators from fully understanding the parents' opinions of their child's math performance. Hence, it remains unclear whether the parent's evaluation was based on the child's real mathematical performance or the parent's own gender stereotype. Finally, most parents in the present study had low education levels. Although no study has revealed that educational level is associated with an individual's intelligence mindset, Claro et al. (2016) reported that students from lower-income families tend to have a fixed mindset. Family income is closely correlated with education level and occupation, all of which constitute socio-economic status (Hanscombe et al., 2012). Based on this, a parent's education level may affect their child's intelligence mindset. Therefore, the results of the present study may not be generalizable to parents with high educational levels. Furthermore, future research should explore whether parents from different socioeconomic statuses influence differences in their child's mindset.

## Data Availability Statement

The raw data supporting the conclusions of this article will be made available by the authors, without undue reservation.

## Ethics Statement

The studies involving human participants were reviewed and approved by College Psychological Health Professional Committee of Hebei Mental Health Association. Written informed consent to participate in this study was provided by the participants' legal guardian/next of kin.

## Author Contributions

FX: data analysis, manuscript draft and revision work. XD: data collection and revision work. XN: data collection. NL: study design and data collection. LZ: data collection, data interpretation and revision work. All authors contributed to the article and approved the submitted version.

## Funding

This work was supported by Chongqing Normal University (20XWB015).

## Conflict of Interest

The authors declare that the research was conducted in the absence of any commercial or financial relationships that could be construed as a potential conflict of interest.

## Publisher's Note

All claims expressed in this article are solely those of the authors and do not necessarily represent those of their affiliated organizations, or those of the publisher, the editors and the reviewers. Any product that may be evaluated in this article, or claim that may be made by its manufacturer, is not guaranteed or endorsed by the publisher.
